# Oxidative stress and anti-oxidant defense system in Iranian women with polycystic ovary syndrome 

**Published:** 2015-06

**Authors:** Mahtab Moti, Leila Amini, Soheila Sadat Mirhoseini Ardakani, Sara Kamalzadeh, Masoomeh Masoomikarimi, Moslem jafarisani

**Affiliations:** 1 *Department of Midwifery, School of Nursing and Midwifery, Iran University of Medical Sciences, Tehran, Iran.*; 2 *Faculty of Nursing and Midwifery, Iran University of Medical Sciences, Tehran, Iran.*; 3 *Department of Biology, College of Basic Sciences, Karaj Branch, Islamic Azad University, Alborz, Iran.*; 4 *Department of Immunology, Torbat Heydariyeh University of Medical Sciences, Torbat Heydariyeh, Iran.*; 5 *Department of Biochemistry, School of Medicine, Shahroud University of Medical Sciences, Shahroud, Iran.*

**Keywords:** *Polycystic ovary syndrome*, *Infertility*, *CRP*, *Antioxidant*, *AOPP*

## Abstract

**Background:**

Polycystic ovary syndrome (PCOS) is a common disorder of infertility which affects more than 100 million women. It is characterized by chronic anovulation, hyper androgenism and obesity. PCOS is also associated with oxidative stress changes.

**Objective:**

Here, we aimed to investigate the level of antioxidants and oxidative stress in Iranian women with PCOS as a predictive factor for cardiovascular disease for the first time in Iran.

**Materials and Methods:**

In this cross sectional study 30 women with PCOS and 30 healthy women were included. C-reactive protein, serum insulin, advanced oxidation protein products, and level of total antioxidants status were measured from blood samples.

**Results:**

The levels of serum insulin, C-reactive protein, advanced oxidation protein productswere significantly increased in women with PCOS compared with healthy women but there was a decrease in level of total antioxidants status in PCOS women.

**Conclusion:**

These changes show that oxidative stress contributes to PCOS and the decrease of antioxidants leads to increase of oxidation products contributing to PCOS.

## Introduction

Polycystic ovary syndrome (PCOS), a heterogeneous disorder, is one of the most common endocrinopathies, which affects 5-10% of women of childbearing age. It is characterized by, obesity, hyperandrogenism, enlarged cystic ovaries, chronic anovulation with oligo-amenorrhea, elevated luteinizing hormone (LH), and infertility (1). Also, insulin resistance is a common property of the syndrome (2). Hyperinsulinemia occurs due to insulin resistance. Independent of obesity, the presence of hyper-insulinemia in women with PCOS has been confirmed (3). PCOS is also associated with an increased number of cardiovascular risk factors, such as oxidative stress, elevated homocysteine (Hcy), and increased C-reactive protein (CRP) it can also affect female fertility (4). Insulin is an atherogenic hormone and hyperinsulinemia may contribute to the development of diabetes, hypertension, and dyslipidemia that is often accompanied by increased total cholesterol and low-density lipoprotein (LDL), triglyceride (TG), and decreased high-density lipoprotein (HDL) levels in PCOS (5). 

Hyperinsulinemia increases ovarian androgen overproduction. Also, dyslipidemia and sex steroids have important influence on cardiovascular diseases (6). Insulin resistance is a cornerstone of cardiovascular risk independent of obesity. Oxidative stress is considered to be one of the major causes of molecular damage to cellular structures and is known to be elevated in patients with diabetes (7). The level of carbonyl content is an oxidative stress marker in plasma proteins. The reduction in the antioxidant status is associated with the development of oxidative stress in diabetes and decreased levels of circulating antioxidants may favor cardiovascular disease (8). 

Advanced oxidation protein products (AOPPs) have been considered as novel markers of oxidant-mediated protein damage and may also act as a novel class of pro- inflammatory mediators. Previous studies suggest a close relation between AOPP and atherosclerosis. PCOS is also associated with an elevated risk of early atherosclerosis (9). Hyperhomocysteinemia has been shown to be an independent risk factor for atherosclerosis (10). All the above-mentioned literature addresses the questions of whether oxidative stress increases in PCOS, and whether there is a relationship between oxidative stress and increased risk of cardiovascular disease in women with PCOS.

This study aimed to determine total antioxidant status, oxidative stress in Iranian women with PCOS for the first time.

## Materials and methods

In this cross sectional study conducted at Sarem Hospital in 2013-2014, thirty PCOS women as the study group (aged 18-38 years) and thirty healthy women as the control group ( aged 16-35 years) were included. The diagnosis of PCOS was based on the presence of oligomenorrhea (fewer than six menstrual periods in the preceding year) with hirsutism (Ferriman- Gallwey score of 7) and multiple subcapsular follicles by transvaginal ultrasound examination (11).

Exclusion criteria included infection diseases, use of medications known to alter insulin secretion or action, and lipoprotein metabolism, hypertension, smoking, family history of cardiovascular disease, and endocrinopathies including diabetes, Cushing syndrome or androgen secreting tumors, thyroid dysfunction, and hyperprolactinemia. Control-group women were healthy volunteers with normal menstrual cycles who had no clinical or biochemical features of hyperandrogenism. This work was approved by Medical Ethics Committee of Shahroud University of Medical Sciences and all participants gave informed consent before the onset of study. Also the groups were matched in terms of body mass index and waist size.

Blood samples were taken after a 10 hr fast and serum glucose, total cholesterol, TG, HDL, very-low-density lipoprotein (VLDL) levels were determined by using commercially available diagnostic kits (Bayer Diagnostics GmbH, Mannheim, Germany) and a Hitachi 917 Automatic Clinical Chemistry Analyzer. LDL levels were calculated by using Friedewald’s formula. Serum C-reactive protein (CRP) was determined by a clinical chemistry system (SPACE, Schiapparelli Biosystems, Woerden, Netherlands), which gives a quantitative result. Waist, hip, body mass index (BMI) measurements were obtained on the study day (on cycle day 2). The BMI was calculated as weight in kilograms divided by the square of height in meters.

The estimate of insulin resistance was calculated by homeostasis model assessment (HOMA) presented by Matthews *et al* (12). Homeostasis model assessment (HOMA-R for insulin resistance and HOMA-F for cell function) is a structural computer model of the glucose-insulin feedback system and performs well in comparison with several tests of insulin sensitivity. Serum CRP was determined turbid metrically (Beckman Coulter, Fullerton, CA). Plasma Hcy levels were measured as total Hcy technique by the high-performance liquid chromatography (HPLC) using Chromo systems kits with fluorescence detector (Roche Diagnostics). The intra- and interassay coefficients of variation were <2%. Serum AOPP levels were determined in plasma by means of using the method devised by Witko-Sarsat *et al* (13). The AOPP concentrations were expressed as mmol/L of chloramine-T equivalents.

Total antioxidant status (TAOS) of plasma samples was assayed by using a commercially available kit (Randox Laboratories, Ltd., Crumlin, UK). The assay principle depends on the inhibition of preformed ABTS (2,2-Azino-di-[3 ethylbenzthiazoline sulfonate]) radical cation by antioxidants present in the plasma. ABTS radical is formed by the incubation of ABTS with a peroxidase (metmyoglobin) and hydrogen peroxide (H_2_O_2_). The results are expressed as mmol/L. Intra-assay and interassay coefficient variation (CV) values were found to be less than 5%. For accuracy, Randox Total Antioxidant Control was used.

**Statistical analysis**

Statistical analysis was made with Student’s *t* test and Pearson correlation analysis. The experimental results are expressed as mean±SD, and the categorical variables were expressed as percentages. Data were analyzed with the SPSS (Statistical Package for the Social Sciences, version 18.0; SPSS, Inc., Chicago, IL) and p<0.05 was considered as significant.

## Results

**Figure 1:**
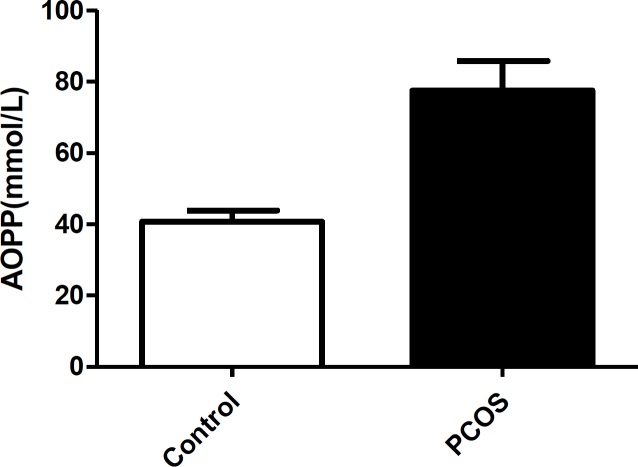
The serum levels of advanced oxidation protein products (AOPPs) in women with polycystic ovary syndrome (PCOS) and control women. Data are shown as mean±SD

The data showed that there were no significant differences in the fasting glucose, mean age, and BMI between the groups. The women with PCOS had significantly higher serum fasting insulin, CRP and LH levels, and higher LH/FSH ratio and HOMA-R than healthy women (for each parameter; p<0.047). However, TAOS was significantly lower in women with PCOS compared with healthy women (p=0.03). Plasma concentrations of AOPP were significantly higher in PCOS patients than in age- and BMI-matched control subjects (79.4±12.0 vs. 45.8±6.1; p=0.01) (Figure 1). Using the mean+2SD (56.2 pg/mL) for AOPP concentrations in the control group as a cut-off value, PCOS patients with AOPP concentrations >56.2 pg/mL were defined as the high-AOPP group (n=30) and those with values <56.2 pg/mL as the normal-AOPP group (n=30).

**Figure 2:**
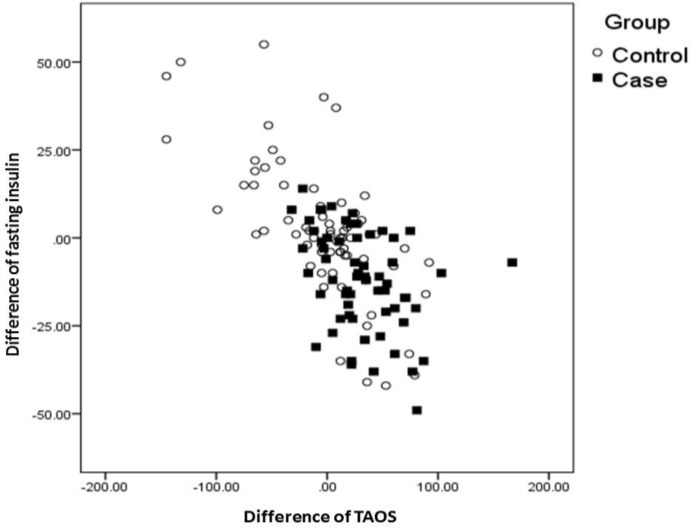
Correlation of the serum levels of fasting insulin and total antioxidant status in women with polycystic ovary syndrome and control women

We then compared clinical and biochemical variables of the PCOS patients in both groups. The mean fasting insulin, HOMA index, Hcy, MDA, total testosterone and CRP were significantly higher in PCOS patients with high AOPP than in those with normal AOPP (Table I). As expected, a positive relationship between fasting insulin levels and HOMA-R was found (r=0.99, p=0.04). TAOS was negatively correlated with fasting insulin levels (r=0.32, p=0.046), HOMA-R (r=0.30, p=047) and CRP levels (r= 0.33, p=0.46) (Figure 2). HDL was inversely associated with fasting insulin levels (r=0.31, p=0.04) and HOMA-R (r=0.28, p=0.048). There were no statistically significant relationships between other parameters. In the Pearson correlation analysis, serum levels of AOPP were positively correlated with fasting insulin (r=0.27; p=0.046), HOMA index (r=0.76; p<0.01) and Hcy (r =0.69; p=0.01), CRP levels (r=0.42; p=0.01). To determine independent factors for plasma concentrations of AOPP, we performed a multiple linear regression in a stepwise manner. In the multiple linear regression analysis, the presence of IR (partial coefficient: b=0.735 [95% CI 0.50-1.5]; p<0.01), fasting insulin (partial coefficient=0.82 [95% CI 0.4-1.4]; p=0.01), and Hcy (partial coefficient: b=0.792 [%95 CI 0.2-1.8]; p=0.01) were independent determinants of plasma AOPP.

**Table I:** Clinical features and metabolic characteristics for healthy women (control group) and PCOS women (study group).

	**Study group ** **(n=30)**	**Control group ** **(n=30)**	**p-value**
Age (years)	26.80 ± 0.67	25.10 ± 0.89	0.73
BMI (kg/m^2^)	24.60 ± 0.90	23.60 ± 1.10	0.44
TAOS (mmol/L)	1.25 ± 0.02	2.13 ± 0.10	0.03
Waist size (cm)	82.20 ± 1.50	81.50 ± 1.20	0.84
Waist/hip ratio	0.81 ± 0.02	0.79 ± 0.01	0.22
Total cholesterol (mg/dL)	172.65 ± 1.10	174.20 ± 1.0	0.67
Triglyceride (mg/dl)	156.30 ± 12.90	148.40 ± 10.30	0.73
LDL-C (mg/dl)	105.50 ± 8.20	96.30 ± 6.70	0.32
HDL-C (mg/dl)	42.30 ± 3.50	56.20 ± 6.30	0.04
Fasting insulin (mIU/min/mL)	17.10 ± 6.10	10.20± 3.40	0.01
Fasting glucose (mg/dL)	86.30 ± 1.10	84.90 ± 0.20	0.64
HOMA index	3.10 ± 1.10	1.70 ± 0.60	0.04
Hcy (mmol/L)	12.80± 1.70	9.30± 1.70	0.001
CRP (mg/dL)	4.90 ± 0.71	1.50 ± 0.69	0.04
LH/FSH ratio	4.63 ± 1.02	2.78 ± 0.98	0.04
FSH (mIU/ml)	4.24 ± 0.52	4.62 ± 0.42	0.23
LH (mIU/ml)	19.63 ± 3.20	12.84 ± 2.10	0.04

Data are shown as mean±SD, and analyzed by student’s *t *test.

BMI: body mass index,

TAOS: total antioxidant status,

LDL: low-density lipoprotein,

HDL: high-density lipoprotein HOMA: homeostasis model assessment,

Hcy: homocysteine,

CRP: C-reactive protein,

FSH: follicle-stimulating hormone

LH: luteinizing hormone

## Discussion

Oxidative stress is an imbalance state between the generation of free radicals which increase the chemical reactivity, and antioxidant defenses modulating the oxidative damages (14). It may cause changes to biological molecules, and these changes accumulate in the biological structures, which may confer molecular damages to cellular and tissue structures (15). Oxidative stress is associated with the pathogenesis of several disease states including aging and aging-related chronic diseases such as diabetes mellitus, atherosclerosis or ischemia reperfusion injury (16).

Although it is not always possible to measure directly in biological systems, numerous biomarkers have been identified providing a measure of oxidative damage to biomolecules (16). Chemical modifications of amino acids lead to introduction of carbonyl groups, which is a consequence of the protein modification by reactive oxygen species (ROS) and contributed to the progression of a number of physiological and pathological disorders. TAOS, described as the ability of serum to eliminate free radical production, consists of multi-compartmental protection against molecular damage of the cell structure. It takes into account the complex interactions that occur between individual antioxidants in vivo.

Numerous detection assays have been identified, one of which is the spectrophotometric approach in which long-lived ABTS radical is detected. Plasma TAOS indicates the concentrations of individual antioxidants. Antioxidants present at high concentrations in the plasma contributing to TAOS. These antioxidants are uric acid, β-carotene, albumin, thiol groups, α-tocopherol, vitamins C and E, and bilirubin (17). TAOS is sensitive to changes in plasma antioxidant levels and oxidative stress status. TAOS, which is an aggregative index of plasma antioxidant status, should be considered in evaluating the effects of oxidative stress in women with PCOS. The oxidant and antioxidant status in women with PCOS have not been extensively studied before.

Although women with PCOS had a markedly higher level of fasting insulin and HOMA-R compared with healthy women, BMI is in normal range in each group. The current study confirms the presence of hyper insulinemia in women with PCOS. C-reactive protein which indicates the enhanced proinflammatory effect accelerates plaque vulnerability and propensity to thrombosis. Elevated serum CRP was reported as a risk factor for the future cardiovascular events (18). There was an increase in CRP level in women with PCOS, and a negative correlation between TAOS and CRP. Present data strengthen the idea that there is an increased risk of cardiovascular events in women with PCOS.

These observations suggest increased oxidative stress and decreased antioxidant levels in PCOS. We found decreased levels of TAOS in women with PCOS, and increased levels of protein carbonyls, conﬁrming free radical attacks on proteins. Our study’s negative correlation between fasting insulin and TAOS, such as, between HOMA-R and TAOS suggests that insulin resistance may have an unfavorable effect on anti-oxidant defense system in PCOS. Also the positive correlation between protein carbonyls and fasting insulin supports the fore mentioned interpretation. As expected, TAOS was inversely associated with protein carbonyls, which may favor many diseases such as cardiovascular disease (19).

High levels of total cholesterol, LDL, and low levels of HDL are the most frequent forms of dyslipidemia, observed in insulin resistance states. Lower HDL levels are another independent of risk factor for cardiovascular events (20). In our study we found a negative correlation between HDL and insulin resistance, in addition to the inverse relationship between HDL and protein carbonyls; however, we could not ﬁnd any statistically signiﬁcant differences in lipid fractions in both groups. Our selection of study participants with same BMI might be a reason.

Our study suggests that increased oxidative stress and decreased antioxidant capacity could contribute to the increased risk of cardiovascular disease in women with PCOS, in addition to the known risk factors such as insulin resistance, hypertension, central obesity, and dyslipidemia. So these patients have to consume anti-oxidant complementary such as vitamin C and vitamin E to achieve an adequate anti-oxidant defense system and reduce the harmful effects of excess of oxidative stress.
